# Supplementary material for: Enhanced stress resilience in potato by deletion of Parakletos
Source: Nat Commun. 2024 Jun 18;15:5224. doi: 10.1038/s41467-024-49584-4 (PMC11189580; doi:10.1038/s41467-024-49584-4)
Supplement: Supplementary file 1 — Supplementary Information [file 41467_2024_49584_MOESM1_ESM.pdf]

Enhanced stress resilience in potato by deletion of *Parakletos*

Zahid *et al.*

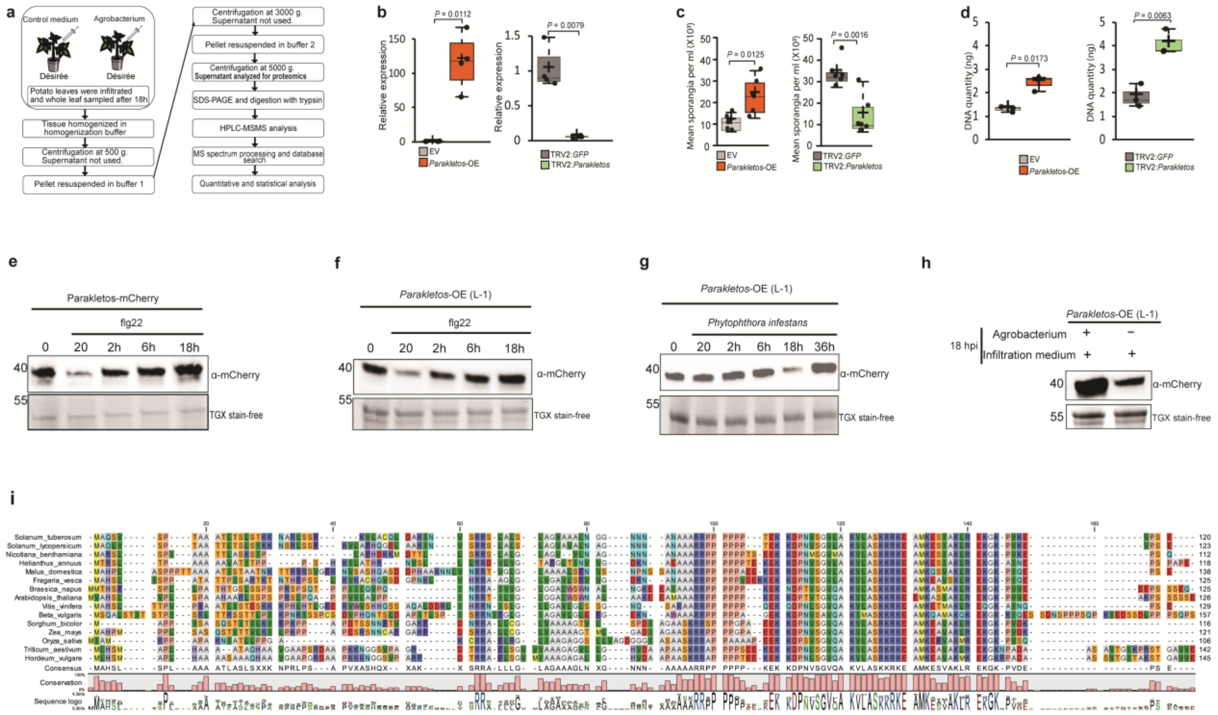

Supplementary Fig. 1. Evaluation of parakletos in plant immune response and allignment information. a, Flow chart for the proteomics experiment. **b-d**, *Parakletos* was screened for involvement in *Phytophthora infestans* resistance by transient expression or silencing in *Nicotiana benthamiana*. **b**, Relative expression of *NbParakletos* in *Parakletos*-OE and *Parakletos*-silenced plants. **c**, Numbers of sporangia per ml in washes from *Parakletos*-OE or silenced plant leaves at 7 dpi. **d**, Relative amounts of *P. infestans* biomass. **e**, Western blot analysis of the *Parakletos-mCherry* fusion protein in *N. benthamiana*, with and without flg22 treatment (1 µM at 0 h, 20 min, 2h, 6h, and 18h). **f**, **g**, **h**, Western blot analyses of the *Parakletos-mCherry* fusion protein in potato (*Parakletos*-OE-L1), with and without flg22 treatment (**f**), after *P. infestans* treatment (**g**), and after *Agrobacterium AGL1* or infiltration medium treatment (**h**), at indicated times. **i**, Amino acid sequence alignment of members of the Parakletos family generated using Clustal Omega. Sequence logo showing amino acid variability and conservation is given below the alignment. The height of the stack is proportional to the conservation of the representative residue. The mature protein starts at amino acid number 68 in the *Solanum tuberosum* protein. Individual data points are plotted as box plots in **b** (n=4), **c** (n=6), and **d** (n=3), n=biological replicates. The centerline in box plots indicate medians, the + sign indicates the mean, the box borders delimit the lower and upper quartiles, and the whiskers show the highest and lowest data points. Data were analysed by two-tailed Student's t-test compared with respective control plants, exact *p* values were shown in figures. Experiments were repeated at least two to three times with similar results. Source data are provided as a Source Data file.

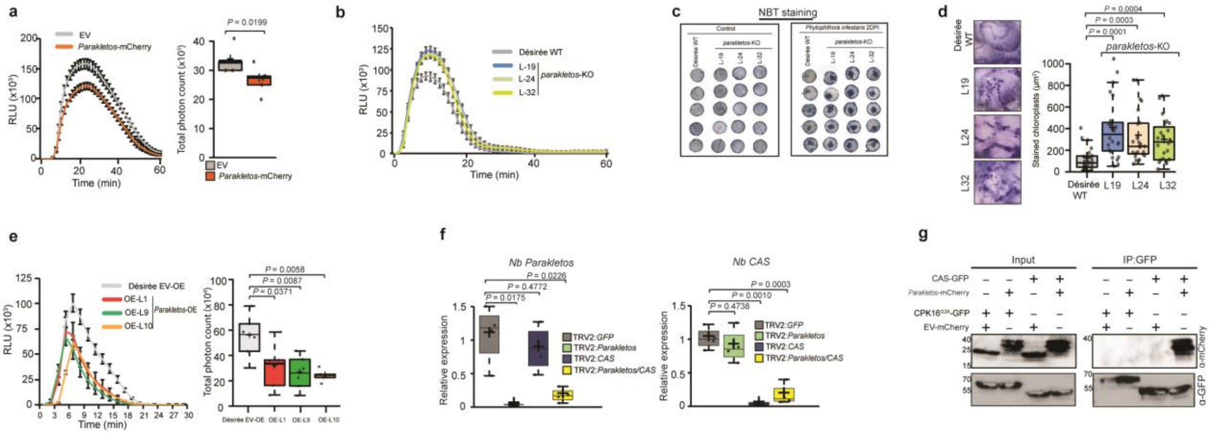

Supplementary Fig. 2. Parakletos involvement in ROS modulation and confirmation of silencing. **a**, Measurement ROS levels and total luminescence in *Nicotiana benthamiana* plants overexpressing Parakletos-mCherry in response to 1 μM flg22 treatment. **b**, ROS production in response to flg22 (8 μM) in *parakletos*-KO (knock-out) potato plants (L19, L24, and L32) compared with Désirée-WT (wild-type control) over 60 minutes. **c**, Visualization of ROS accumulation using nitroblue tetrazolium (NBT) staining in *parakletos*-KO and Désirée-WT (control) potato plants, with and without *Phytophthora infestans* infection at 2 days post-infection. **d**, Microscopic examination of NBT-stained leaf sections at 400× magnification; quantification of the stained chloroplast area was performed with FIJI (ImageJ) and is represented in a box plot. **e**, Monitoring of ROS levels in response to flg22 (8 μM) in *Parakletos*-OE (overexpression) potato plants (OE-L1, OE-L9, OE-L10) and calculation of total luminescence over 30 minutes. **f**, Expression levels of *NbParakletos* and *NbCAS* in *N. benthamiana* plants to confirm dual silencing, with transcript levels normalized to *EF-1α*. **g**, Co-immunoprecipitation analysis of CASGFP/CPK16^{G2A}-GFP and mCherry/*Parakletos*-mCherry after co-expression in *N. benthamiana*. Plant tissues were collected two days post *Agrobacterium* infiltration for immunoprecipitation with GFP agarose beads. Individual data points are plotted as box plots in **a,e** (n=6), **d** (n=24), and **f** (n=4), n=biological replicates. The centerline in box plots indicate medians, the + sign indicates the mean, the box borders delimit the lower and upper quartiles, and the whiskers show the highest and lowest data points. Data were analysed by two-tailed Student's t-test compared with respective control plants, exact *p* values were shown in figures. Experiments were repeated at least two to three times with similar results. Source data are provided as a Source Data file.

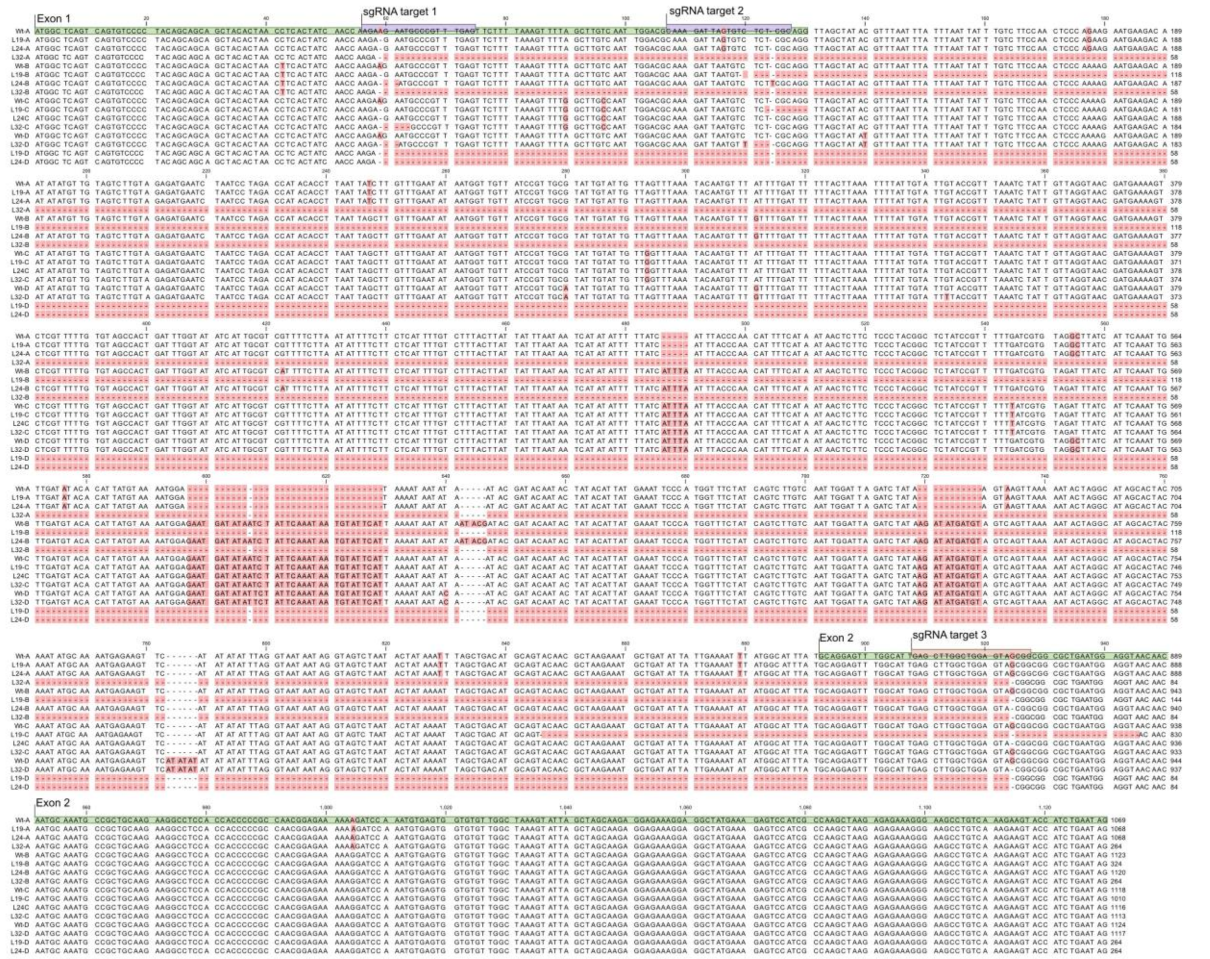

Supplementary Fig. 3. Confirmation of *parakletos*-KO lines in potato. Sequence alignment of all four alleles of the Parakletos gene in wild type (WT) plants, labeled as Wt-A, Wt-B, Wt-C, and Wt-D. This is compared with the alleles from the *parakletos-KO* lines (L19, L24, and L32), highlighting mutations in all alleles. The alignment was generated using Clustal Omega. Annotations on the sequence indicate the locations of exon 1 and exon 2, as well as the target sites for the single-guide RNA (SgRNA).

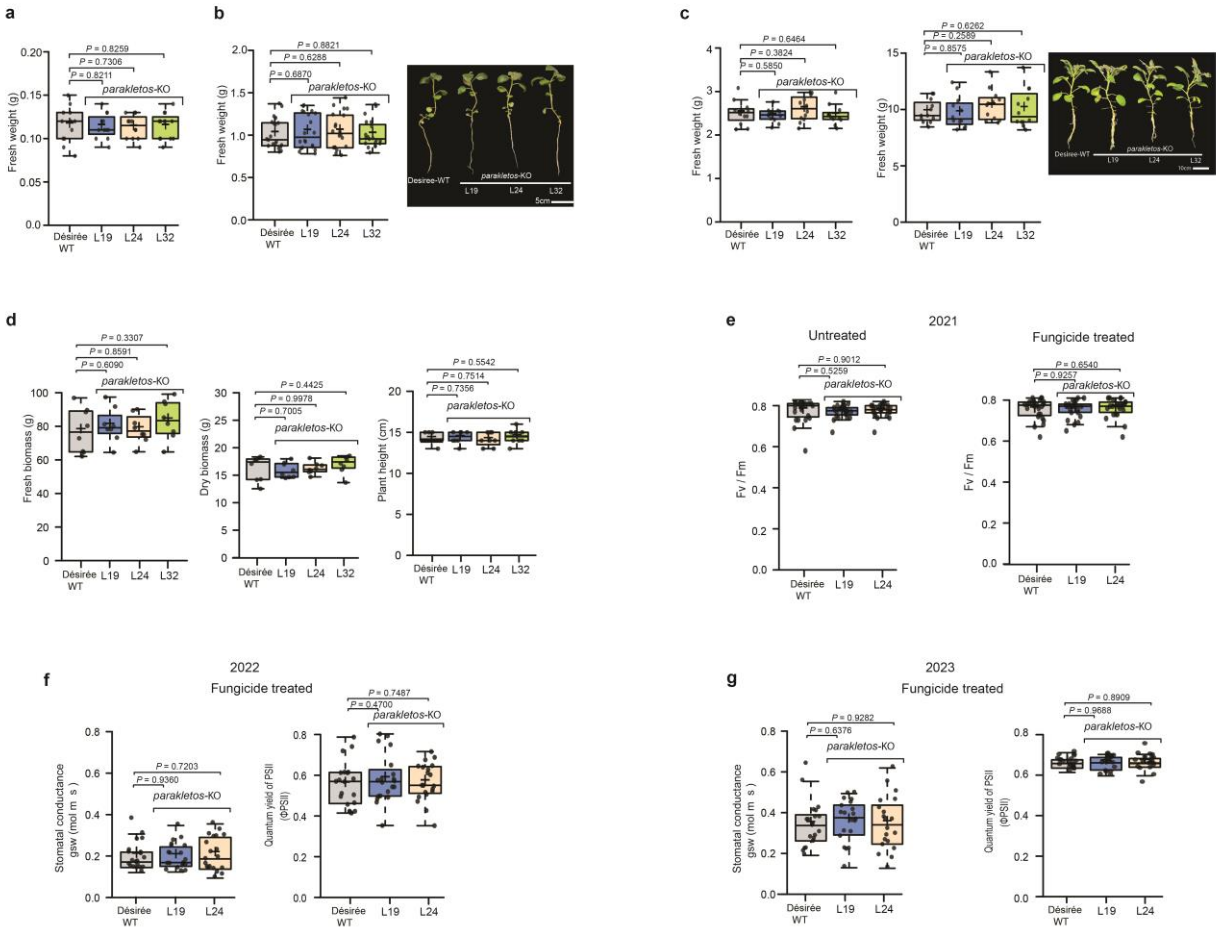

Supplementary Fig. 4. ***parakletos*-KO potato plant showed no negative affect on plant growth and physiological parameters**. **a-c**, Control experiments for abiotic stress. **a**, Fresh weight of seeding (rooting plants) after 7 days under in vitro conditions, prior to growth in hydroponic conditions for both salt and drought stress. **b**, Fresh weight of the plant after 7 days under hydroponic conditions, serving as a control for the salt stress experiment. **c**, Left panel: fresh weight of the plant after 14 days under hydroponic conditions, before the application of drought stress. Right panel: Fresh weight of the plant after 23 days under hydroponic conditions, with an image of the plant at the conclusion of the drought stress experiment. **d**, Comparison of plant heights, fresh weights, and dry weights between wild-type and *Parakletos*-KO plants at 5 weeks, conducted under controlled growth conditions. **e**, Evaluation of maximum quantum yield of PSII (Fv/Fm) in untreated and fungicide-treated plots during the 2021 field trials, measured with the FluorPen FP 100 instrument. **f**, **g**, Measurements of stomatal conductance (gsw, mol m^{-2} s^{-1}) and quantum yield of PSII (ΦPSII), taken with the LI-COR (LI-600) instrument during the 2022 and 2023 field trials. Individual data points are plotted for each parameter (**a**, n=12; **b**, n=20; **c** left panel, n=12; **c** right panel, n=12; **d**, n=8; **e**, n=24; **f**, **g**, n=20). The centerline in box plots indicate medians, the + sign indicates the mean, the box borders delimit the lower and upper quartiles, and the whiskers show the highest and lowest data points. Data were analysed by two-tailed Student's t-test compared with respective control plants; exact p values were shown in the figures. Experiments were repeated at least two to three times with similar results (**a-d**). Source data are provided as a Source Data file.

Supplementary Table 1. List of primers.

Primer name	Sequence	Used for
oCmYLCV	TGCTCTTCGCGCTGGCAGACATACTGTCC CAC	Vector constructs
CSY_7A	TCGTCTCCGCCCGTTTGAGTCTGCCTATA CGGCAGTGAAC	
REP_7A	TCGTCTCAGGGCATTCTTCTGTTTTAGAG CTAGAAATAGC	
CSY_7B	TCGTCTCCCATTAATCTTTGCTGCCTATAC GGCAGTGAAC	
REP_7B	TCGTCTCAAATGTCTCTCGCGTTTTAGAG CTAGAAATAGC	
CSY_7C	TCGTCTCCCCAGCCAAGCTCCTGCCTATA CGGCAGTGAAC	
REP_7C	TCGTCTCATTCTCCGTTGGCGTTTTAGAG CTAGAAATAGC	
CSY_term	TGCTCTTCTGACCTGCCTATACGGCAGTG AAC	
Guide 1	AGAAGAATGCCCGTTTGAG	
Guide 2	CAAAGATTAATGTCTCTCGC	
Guide 3	GAGCTTGGCTGGAGTAGCGG	
attB1-Nb-Parakletos_F	GGGGACAAGTTTGTACAAAAAAGCAGGC TATGGCTCGGTCATTGTCTCC	For Parakletos-OE
attB1-Nb-Parakletos_R	GGGGACCACTTTGTACAAGAAAGCTGGG TCTATTGAGATGGTTCTTTGACTGGC	
Nb-Parakletos_OE_qPCR_F	CGGAGTTGTGCTAAATGCAG	qPCR for Parakletos-OE
Nb-Parakletos_OE_qPCR_R	CCAAGCTAAGGGAGAAAGGG	
TRV2-Nb-Parakletos_F	GGCTACGGTCTCCATTCTTGAGATGGTTC TTTGACTGG	For Parakletos-VIGS
TRV2-Nb-Parakletos_F	GGCTACggtctcgatccGCTCGGTCATTGTCTC CA	
Nb-Parakletos_VIGS_qPCR_F	TGAGTGGTGTCATGGCTAAAG	qPCR for Parakletos-VIGS
Nb-Parakletos_VIGS_qPCR_R	AGAAGAGCAAGCATGCAACA	
NbCAS_F	TGCGAAGAAAGTGGAAGCTG	qPCR
NbCAS_R	GCTTACTCTGCAACCAACCC	
F NF2	GGGTATTTTGTAGCCTTTGGTGT	Screening mutant
St72_R2e (Parakletos)	CTTTAGCCAACACACCACTCAC	
St72_NF3 (Parakletos)	TGTGGTACGTGTTCGATCTTGAC	
St72 _R4e (Parakletos)	TCGCCAAGCTAAGAGAGAA	
St72 _NR3 (Parakletos)	AAGTCTCAAGGTTTTAAAGGAAGGA	
St72 _NR4 (Parakletos)	AACCAATCATAAAAGCTATTCATTTAT	
St72_F2e (Parakletos)	CCCTACAGCAGCAGCTACAC	
St72-A3-nF2 (Parakletos)	ACCAATAAAATTTGAATGCCAAC	
