# Supplementary material for: Enhanced stress resilience in potato by deletion of Parakletos
Source: Nat Commun. 2024 Jun 18;15:5224. doi: 10.1038/s41467-024-49584-4 (PMC11189580; doi:10.1038/s41467-024-49584-4)
Supplement: Supplementary file 3 — Description of Additional Supplementary Files [file 41467_2024_49584_MOESM3_ESM.pdf]

Description of Additional Supplementary File

File name: Supplementary Data 1

Description: List of proteins significantly ($p < 0.05$) differentially regulated relative to control
