# Supplementary material for: Enhanced stress resilience in potato by deletion of Parakletos
Source: Nat Commun. 2024 Jun 18;15:5224. doi: 10.1038/s41467-024-49584-4 (PMC11189580; doi:10.1038/s41467-024-49584-4)
Supplement: Supplementary file 5 — Reporting Summary [file 41467_2024_49584_MOESM5_ESM.pdf]

nature portfolio

Corresponding author(s): Erik Andreasson

Last updated by author(s): Nov 11, 2023

Reporting Summary

Nature Portfolio wishes to improve the reproducibility of the work that we publish. This form provides structure for consistency and transparency in reporting. For further information on Nature Portfolio policies, see our Editorial Policies and the Editorial Policy Checklist.

Statistics

For all statistical analyses, confirm that the following items are present in the figure legend, table legend, main text, or Methods section.

n/a	Confirmed	
☐	☒	The exact sample size (*n*) for each experimental group/condition, given as a discrete number and unit of measurement
☐	☒	A statement on whether measurements were taken from distinct samples or whether the same sample was measured repeatedly
☐	☒	The statistical test(s) used AND whether they are one- or two-sided<br>*Only common tests should be described solely by name; describe more complex techniques in the Methods section.*
☒	☐	A description of all covariates tested
☒	☐	A description of any assumptions or corrections, such as tests of normality and adjustment for multiple comparisons
☐	☒	A full description of the statistical parameters including central tendency (e.g. means) or other basic estimates (e.g. regression coefficient) AND variation (e.g. standard deviation) or associated estimates of uncertainty (e.g. confidence intervals)
☐	☒	For null hypothesis testing, the test statistic (e.g. *F*, *t*, *r*) with confidence intervals, effect sizes, degrees of freedom and *P* value noted<br>*Give P values as exact values whenever suitable.*
☒	☐	For Bayesian analysis, information on the choice of priors and Markov chain Monte Carlo settings
☒	☐	For hierarchical and complex designs, identification of the appropriate level for tests and full reporting of outcomes
☒	☐	Estimates of effect sizes (e.g. Cohen's *d*, Pearson's *r*), indicating how they were calculated

Our web collection on statistics for biologists contains articles on many of the points above.

Software and code

Policy information about availability of computer code

Data collection	Proteomics data are deposed to the ProteomeXchange Consortium (http://proteomecentral.proteomexchange.org) via the PRIDE partner repository with the accession number PXD038421.
Data analysis	Raw MS data were converted to Mascot generic file (mgf) format with ProteoWizard. S. tuberosum protein database from UniProt (www.uniprot.org), that was concatenated with a decoy database of equal size (random protein sequences with conserved protein length and amino acid distribution: 106,210 target and decoy protein entries in total) was generated using a modified version of the decoy.pl script from MatrixScience (http://www.matrixscience.com/help/decoy_help.html). This database was then searched using the mgf files with Mascot version 2.3.01 in the Proteios software environment (https://proteios.org). Search results were exported from Mascot as XML files, including query level results, with a modification of the export script to include protein accession numbers, and the query (spectrum) level results, which were then imported into Proteios. MS1 peptide features were detected using Dinosaur (https://github.com/fickludd/dinosaur), while the other data processing steps were performed in Proteios. The resulting peptide data were normalized using Loess-G normalization in Normalyzer software (http://quantitativeproteomics.org/normalyzer). The normalized peptide data were imported into InfernoRDN (https://omics.pnl.gov/software/InfernoRDN) and protein intensities were generated using the RRollup procedure. Differential expression was statistically analyzed using Limma. The ShinyGO v0.60: KEGG Enrichment Analysis program (50) was used to detect indications of pathway involved in our proteomic datasets.

For manuscripts utilizing custom algorithms or software that are central to the research but not yet described in published literature, software must be made available to editors and reviewers. We strongly encourage code deposition in a community repository (e.g. GitHub). See the Nature Portfolio guidelines for submitting code & software for further information.

Data

Policy information about availability of data

All manuscripts must include a data availability statement. This statement should provide the following information, where applicable:

- Accession codes, unique identifiers, or web links for publicly available datasets
- A description of any restrictions on data availability
- For clinical datasets or third party data, please ensure that the statement adheres to our policy

All data supporting the findings of this study are available within the paper and its Supplementary Information, except the proteomics data araw data that are deposed to the ProteomeXchange Consortium (http://proteomecentral.proteomexchange.org) via the PRIDE partner repository with the accession number PXD038421.

Research involving human participants, their data, or biological material

Policy information about studies with human participants or human data. See also policy information about sex, gender (identity/presentation), and sexual orientation and race, ethnicity and racism.

Reporting on sex and gender	N/A
Reporting on race, ethnicity, or other socially relevant groupings	N/A
Population characteristics	N/A
Recruitment	N/A
Ethics oversight	N/A

Note that full information on the approval of the study protocol must also be provided in the manuscript.

Field-specific reporting

Please select the one below that is the best fit for your research. If you are not sure, read the appropriate sections before making your selection.

☒ Life sciences ☐ Behavioural & social sciences ☐ Ecological, evolutionary & environmental sciences

For a reference copy of the document with all sections, see nature.com/documents/nr-reporting-summary-flat.pdf

Life sciences study design

All studies must disclose on these points even when the disclosure is negative.

Sample size	Sample sizes were chosen based on previous publications or preliminary tests. Previous publications considered to determine the sample size include:<br>Gene expression analysis (DOI: 10.1105/tpc.16.00650);<br>ROS burst (DOI: 10.1105/tpc.16.00650);<br>Plant infection assays (DOI: 10.1016/j.cell.2017.06.008; DOI: 10.1038/s41587-019-0267-z; DOI: 10.1038/s41477-022-01327-3);<br>Agronomic traits (doi.org/10.1007/s10658-010-9582-4).
Data exclusions	No data were excluded.
Replication	All experiments were successfully repeated at least two or three times as indicated, and the number of independent experiments or biological replicates is indicated in the figure legends.
Randomization	All samples were arranged randomly into experimental groups. Plants of the treatment and control groups were grown side by side randomly to minimize unexpected environmental variations during growth and experimentation.
Blinding	Investigators were not blinded to the allocation in the experiments, which do not contain clinical trials. In addition, the research materials are edited plants, which need to be strictly regulated and clearly labeled during the experimental process, so the blinding design is not applicable to this system.

Reporting for specific materials, systems and methods

We require information from authors about some types of materials, experimental systems and methods used in many studies. Here, indicate whether each material, system or method listed is relevant to your study. If you are not sure if a list item applies to your research, read the appropriate section before selecting a response.

Materials & experimental systems

n/a	Involved in the study
☐	☒ Antibodies
☒	☐ Eukaryotic cell lines
☒	☐ Palaeontology and archaeology
☒	☐ Animals and other organisms
☒	☐ Clinical data
☒	☐ Dual use research of concern
☐	☒ Plants

Methods

n/a	Involved in the study
☒	☐ ChIP-seq
☒	☐ Flow cytometry
☒	☐ MRI-based neuroimaging

Antibodies

Antibodies used	BIO-RAD AHP2326, Polyclonal IgG Goat anti mCherry<br>polyclonal rabbit anti-goat immunoglobulins/HRP (DAKO) 2717114<br>GFP Monoclonal antibody 3E& (invitrogen) A-11120
Validation	AHP2326: https://www.bio-rad-antibodies.com/polyclonal/mcherry-antibody-ahp2326.html?f=purified<br><br>2717114: https://www.agilent.com/en/product/specific-proteins/elisa-kits-accessories/rabbit-anti-goat-immunoglobulins-hrp-affinity-isolated-2717114#productdetails<br><br>A-11120 https://www.thermofisher.com/antibody/product/GFP-Antibody-clone-3E6-Monoclonal/A-11120

Dual use research of concern

Policy information about dual use research of concern

Hazards

Could the accidental, deliberate or reckless misuse of agents or technologies generated in the work, or the application of information presented in the manuscript, pose a threat to:

No	Yes
☒	☐ Public health
☒	☐ National security
☒	☐ Crops and/or livestock
☒	☐ Ecosystems
☒	☐ Any other significant area

Experiments of concern

Does the work involve any of these experiments of concern:

No	Yes
☒	☐ Demonstrate how to render a vaccine ineffective
☒	☐ Confer resistance to therapeutically useful antibiotics or antiviral agents
☒	☐ Enhance the virulence of a pathogen or render a nonpathogen virulent
☒	☐ Increase transmissibility of a pathogen
☒	☐ Alter the host range of a pathogen
☒	☐ Enable evasion of diagnostic/detection modalities
☒	☐ Enable the weaponization of a biological agent or toxin
☒	☐ Any other potentially harmful combination of experiments and agents

Plants

Seed stocks

Solanum tuberosum cv Désirée has been used.

Novel plant genotypes

The coding sequence of the potato Parakletos gene was analyzed for possible CRISPR targets and their numbers of off-targets using CRISPOR (http://crispor.tefor.net). The gRNA spacers were assembled into the Csy4 multi-gRNA vector pDIRECT_22C, to form the plasmid pDIRECT_22C_StParakletos. The vector was Sanger-sequenced, then potato parakletos-KO mutant lines were created by Agrobacterium tumefaciens C58 carrying pDIRECT_22C_dmr6-1. Leaf explants were incubated under reduced light (50% intensity) for 48 h before they were transferred to selective media (400 mg/L cefotaxime + 100 mg/L kanamycin, and 2 mg/L and 5 mg/L zeatin ribose) for regeneration. Leaf explants were sub-cultured onto fresh media every 7–10 d to maintain selection pressure. Shoots that emerged after 4–5 weeks were dissected and rooted on MS media containing no plant growth regulators but with continued selection (100 mg/L kanamycin). Only shoots that initiated roots in the selective media were screened at the molecular level.

Authentication

Describe any authentication procedures for each seed stock used or novel genotype generated. Describe any experiments used to assess the effect of a mutation and, where applicable, how potential secondary effects (e.g. second site T-DNA insertions, mosiacism, off-target gene editing) were examined.
